# Highly Efficient Room‐Temperature Spin‐Orbit‐Torque Switching in a Van der Waals Heterostructure of Topological Insulator and Ferromagnet

**DOI:** 10.1002/advs.202400893

**Published:** 2024-03-22

**Authors:** Gyu Seung Choi, Sungyu Park, Eun‐Su An, Juhong Bae, Inseob Shin, Beom Tak Kang, Choong Jae Won, Sang‐Wook Cheong, Hyun‐Woo Lee, Gil‐Ho Lee, Won Joon Cho, Jun Sung Kim

**Affiliations:** ^1^ Department of Physics Pohang University of Science and Technology Pohang 37673 Republic of Korea; ^2^ Center for Artificial Low Dimensional Electronic Systems Institute for Basic Science (IBS) Pohang 37673 Republic of Korea; ^3^ Center for Complex Phase of Materials Max Planck POSTECH/Korea Research Initiative Pohang 37673 Republic of Korea; ^4^ Laboratory for Pohang Emergent Materials Department of Physics, POSTECH Pohang 37673 Republic of Korea; ^5^ Rutgers Center for Emergent Materials and Department of Physics and Astronomy Rutgers University Piscataway NJ 08854 USA; ^6^ Device Research Center Samsung Advanced Institute of Technology (SAIT) Samsung Electronics Co., Ltd 130 Samsung‐ro, Yeongtong‐gu, Suwon‐si Gyeonggi‐do 16678 Republic of Korea

**Keywords:** 2D ferromagnet, charge‐to‐spin conversion, spin‐orbit‐torque, topological insulator, Van der Waals heterostructure

## Abstract

All‐Van der Waals (vdW)‐material‐based heterostructures with atomically sharp interfaces offer a versatile platform for high‐performing spintronic functionalities at room temperature. One of the key components is vdW topological insulators (TIs), which can produce a strong spin‐orbit‐torque (SOT) through the spin‐momentum locking of their topological surface state (TSS). However, the relatively low conductance of the TSS introduces a current leakage problem through the bulk states of the TI or the adjacent ferromagnetic metal layers, reducing the interfacial charge‐to‐spin conversion efficiency (*q*_ICS_). Here, a vdW heterostructure is used consisting of atomically‐thin layers of a bulk‐insulating TI Sn‐doped Bi_1.1_Sb_0.9_Te_2_S_1_ and a room‐temperature ferromagnet Fe_3_GaTe_2,_ to enhance the relative current ratio on the TSS up to ≈20%. The resulting *q*_ICS_ reaches ≈1.65 nm^−1^ and the critical current density *J*_c_ ≈0.9 × 10^6^ Acm^−2^ at 300 K, surpassing the performance of TI‐based and heavy‐metal‐based SOT devices. These findings demonstrate that an all‐vdW heterostructure with thickness optimization offers a promising platform for efficient current‐controlled magnetization switching at room temperature.

## Graphical abstract

Current‐driven magnetization switching via spin‐orbit torque is achieved at room temperature in a van der Waals heterostructure of a bulk‐insulating topological insulator Sn‐doped Bi_1.1_Sb_0.9_Te_2_S_1_ and a room temperature ferromagnet, Fe_3_GaTe_2_. By controlling the thickness of the constituent layers and maximizing the relative current ratio on the topological surface states, the highly efficient spin‐orbit‐torque operation is realized, surpassing the performance of most previous devices.

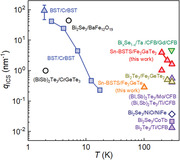

## 1 Introduction

Energy efficient current‐induced magnetization switching at room temperature using strong charge‐to‐spin conversion and effective spin‐orbit‐torque (SOT) operation is one of key challenges for next‐generation spintronic applications.^[^ ^1^, ^2^ ^]^ In contrast to conventional heavy metals (HMs), topological insulators (TIs) have shown high charge‐to‐spin conversion efficiency, mainly stemming from the current‐induced spin polarization of topologically‐protected spin‐momentum locking surface states.^[^ ^3^, ^4^ ^]^ Among various TIs, bismuth antimony trichalcogenides (Bi,Sb)_2_ *Ch*_3_ (*Ch* = S, Se, and Te) and their derivatives have emerged as the most promising material platform due to their large bulk gap of ≈0.3 eV, a simple TSS having a single Dirac cone with helical spin texture, and van der Waals (vdW) layered structure, which are favorable for suppressing the bulk contribution, enhancing charge‐to‐spin conversion, and realizing atomically clean interface for high spin transparency, respectively.^[^ ^5^, ^6^, ^7^, ^8^, ^9^, ^10^, ^11^, ^12^, ^13^, ^14^, ^15^ ^]^ Various types of ferromagnet (FM) layers have been deposited on top of the (Bi,Sb)_2_ *Ch*_3_‐type TI layers for the SOT devices, demonstrating more efficient SOT operation compared to the HM/FM devices.^[^ ^16^, ^17^, ^18^, ^19^, ^20^ ^]^ However, all‐vdW TI/FM heterostructure devices, in which strong charge‐to‐spin conversion and high interfacial spin transparency can be achieved simultaneously at room temperature, have rarely been studied, partly due to the lack of suitable vdW FMs.

For room‐temperature SOT operation in all‐vdW TI/FM heterostructure devices, FM vdW materials need to possess several properties including strong perpendicular magnetic anisotropy (PMA) for efficient SOT from the spin injection from the TSS, and high Curie temperature (*T*_c_) above room temperature, and metallic conduction for effective electrical spin detection by anomalous Hall effect (AHE) or tunneling magnetoresistance.^[^ ^21^, ^22^ ^]^ One of the promising candidates satisfying these conditions is Fe_3_GaTe_2_ (*T*_c_ = 350 K). Successful SOT operations using Fe_3_GaTe_2_ and its sister compound Fe_3_GeTe_2_ (*T*_c_ = 220 K) have been demonstrated in their heterostructures with deposited HMs, but they exhibit low SOT efficiency, necessitating high current density for magnetization switching.^[^ ^23^, ^24^, ^25^, ^26^ ^]^ Recently a vdW heterostructure of Fe_3_GeTe_2_ and the topological semimetal WTe_2_ has exhibited relatively high SOT efficiency but well below room temperature,^[^ ^27^, ^28^, ^29^ ^]^ and MBE‐grown Fe_3_GeTe_2_/Bi_2_Te_3_ films demonstrate possible SOT operation at room temperature, but with a tiny remnant magnetization and negligible hysteresis due to weak interfacial ferromagnetism induced by the proximity effect.^[^ ^30^ ^]^ In such TI/FM based vdW SOT devices, a challenge lies in the substantial mismatch of conductivities between the two constituent TI and FM layers, as well as between the bulk states and the TSSs within the TI layers. At room temperature, the current leakage to the trivial bulk states of the TI layers or to the FM layers significantly reduces the effective current density through the TSS of TI layers and consequently the SOT efficiency. Here using high‐quality Sn‐doped Bi_1.1_Sb_0.9_Te_2_S (Sn‐BSTS) and Fe_3_GaTe_2_ layers and controlling their thickness, we demonstrate efficient SOT operation with a high charge‐to‐spin efficiency and a low critical switching current at room temperature, surpassing the performances of HM/Fe_3_(Ga,Ge)Te_2_ devices^[^ ^23^, ^24^, ^25^, ^26^ ^]^ and other TI/FM devices.^[^ ^16^, ^17^, ^20^, ^30^, ^31^ ^]^

## 2 Results

**Figure 1:**
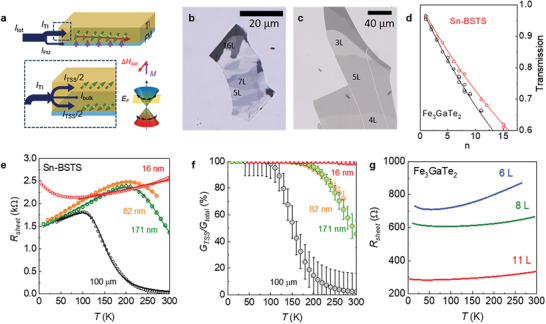
Device configuration and characterization of Sn‐BSTS and Fe_3_GaTe_2_ nanodevices. a) A schematic of a TI/FM heterostructure illustrating the current distribution inside the device. The applied electrical current divides between the FM and TI layers. Inside the TI layers, the current further splits between the bulk state and the TSSs at the top and bottom surfaces. b,c) Transmitted optical images of thin flakes of Sn‐BSTS (b) and Fe_3_GaTe_2_ (c) exfoliated on the deposited Al_2_O_3_ layers on top of a sapphire substrate. The number of layers is labeled in the images of nanoflakes. d) Layer‐dependent optical transmission *G* normalized by that of the substrate (*G*_0_) for Sn‐BSTS and Fe_3_GaTe_2_ nanoflakes. The black line represents the best fit to the Beer‐Lambert law. e) Temperature‐dependent sheet resistance *R*_sheet_(*T*) for Sn‐BSTS samples with different thicknesses (*d*) of 100 µm, 171 nm, 82 nm, and 16 nm. The solid lines are the best fits to the parallel conduction model. For *d =* 16 nm, the fit was done for high temperature data except for the low‐temperature upturn due to localization effect. f) The corresponding ratio between conductance of the TSS (*G*_TSS_) and the total conductance (*G*_tot_) as a function of temperature. g) Temperature‐dependent sheet resistance *R*_sheet_(*T*) for Fe_3_GaTe_2_ samples with different thicknesses of Fe_3_GaTe_2_ 6, 8, and 11 layers.

**Figure** **1**a schematically shows the current distribution in TI/FM heterostructure and the SOT mechanism acting on the magnetization of the adjacent FM layers. When an electric current flows in the TI layers, spin current can be generated from the three channels, the topological surface state (TSS), the Rashba surface states, and the bulk states of TIs. It has been known that the TSS dominantly contribute to spin current generation, while the bulk states contribute negligibly due to their small spin Hall effect.^[^ ^20^, ^32^ ^]^ The Rashba surface states with opposite spin texture to that of the TSS would contribute negatively to spin current.^[^ ^33^, ^34^, ^35^ ^]^ Previous studies have clearly suggested that to maximize the current through the TSS, leakage to the bulk states of TIs should be minimized, which requires precise tuning of the Fermi level within the bulk gap, so making TI layers bulk‐insulating with low conductance.^[^ ^2^, ^20^, ^36^, ^37^, ^38^, ^39^, ^40^, ^41^ ^]^ However, using bulk‐insulating TI layers inevitably leads to current leakage into the ferromagnetic metal layers, which have a sheet conductance more than an order of magnitude larger than that of TIs.^[^ ^3^, ^4^, ^42^, ^43^ ^]^ Assuming the same thickness of TI and FM layers with their bulk conductivities, less than 1% of current can flow through the TI layers. This current shunting problem is one of the issues that reduce the charge‐to‐spin conversion, and thus the resulting SOT in TI‐based vdW SOT devices.

To fully utilize the TSS states of TI layers and intrinsic ferromagnetism with a significant PMA of FM layers, we employed a vdW heterostructure comprising atomically‐thin Sn‐BSTS layers and Fe_3_GaTe_2_ layers. Achieving precise thickness control for both Sn‐BSTS and Fe_3_GaTe_2_ is crucial, but conventional exfoliation methods face challenges in isolating atomically thin layers of these materials in a reasonable size because of the insufficient intralayer bonding. To overcome this, we utilized the recently developed Al_2_O_3_‐assisted exfoliation method,^[^ ^44^ ^]^ a dry transfer technique for flipping the exfoliated crystals, and a focused ion beam micro‐machining, which allow us to successfully fabricate the heterostructure devices (see Methods and Supplementary Figure S1, Supporting Information). These methods, essential for the production of our Sn‐BSTS/Fe_3_GaTe_2_ heterostructure, are applicable to a broad range of vdW crystal heterostructures with atomically thin layers unattainable conventional exfoliation methods. The optical images after Al_2_O_3_‐assisted exfoliation are shown in Figure 1b,c for Sn‐BSTS and Fe_3_GaTe_2_ respectively, whose thickness is estimated by the optical contrast (Figure 1d) and atomic force microscopy (Supplementary Figure S5, Supporting Information)

Sn‐BSTS is the most bulk‐insulating TI known to date,^[^ ^45^ ^]^ allowing us to maximize its TSS contribution to the current within the TI layers. The temperature‐dependent sheet resistance *R*_sheet_(*T*) of a bulk Sn‐BSTS exhibits a semiconducting behavior at high temperatures, followed by a metallic behavior with lowering temperature below a crossover temperature ≈100 K (Figure 1e), consistent with the previous studies.^[^ ^46^ ^]^ This *R*_sheet_(*T*) behavior can be well explained by the parallel conduction model involving the semiconducting bulk state and the metallic TSS,^[^ ^47^, ^48^, ^49^ ^]^ which is described by $R_{\mathrm{sheet}}=\frac{1}{G_{\mathrm{tot}}}\;=\frac{1}{G_{\mathrm{bulk}}+G_{\mathrm{TSS}}}\;$ with $G_{\mathrm{bulk}}=\frac{t}{\rho_{b}e^{\Delta /kT}}\;$ and $G_{\mathrm{TSS}}=\frac{1}{R_{0}+AT}\;$, where *k* is Boltzmann constant, ρ_b_ is high temperature bulk resistivity, Δ is the activation energy, *R*_0_ is the residual resistance of the TSS, and *A* is the coefficient capturing electron‐phonon scattering in the TSS. The best fit to this model accurately reproduces the experimental results, yielding the relative ratio of the conduction through the TSS, *G*_TSS_/*G*_tot_ (Figure 1f). Exfoliated flakes with thickness ≈100 nm exhibit the similar *R*_sheet_(*T*) behavior with a higher crossover temperature of ≈200 K, which shifts to above room temperature with further lowering the thickness down to ≈16 nm. Accordingly, the relative conductance ratio of the TSS against the bulk *G*_TSS_/*G*_total_ (*T*), estimated from the parallel conduction model, indicates that the upper bound of the temperature range for the TSS‐dominant conduction (*G*_TSS_/*G*_total_ > 0.9) extends up to room temperature. We note that further reduction of the thickness of Sn‐BSTS down to several layers can induce hybridization of the top and bottom TSS, suppressing their helical spin texture. We thus used the Sn‐BSTS flakes with thickness of 10‐40 nm for the SOT devices presented below.

**Figure 2:**
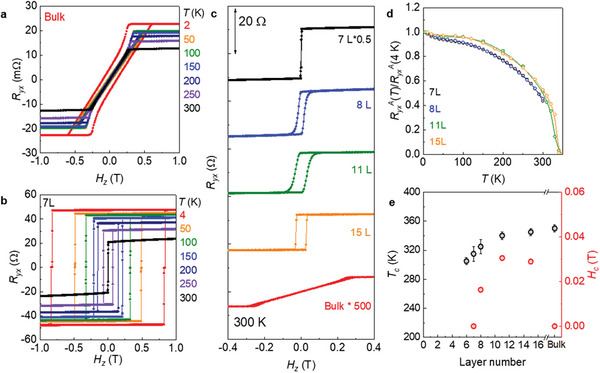
Magnetic properties of Fe_3_GaTe_2_ bulk crystal and atomically‐thin flakes. a,b) Magnetic‐field‐dependent Hall resistance *R_yx_* (*H*) at various temperatures for a Fe_3_GaTe_2_ bulk crystal (a) and flake with thickness of 7 layer (7L). c) Magnetic‐field‐dependent Hall resistance *R_yx_* (*H*) taken at 300 K for Fe_3_GaTe_2_ flakes with different thicknesses. The data are vertically shifted for clarity. d) Temperature‐dependent anomalous Hall resistance *R_yx_^A^* (*T*) at zero magnetic field, normalized by *R_yx_^A^* (*T* = 4 K), for Fe_3_GaTe_2_ flakes with different thicknesses. e) Thickness dependent critical temperature *T*_c_ and coercive field *H*_c_ at 300 K.

To minimize the current flow of the FM metallic layers, we need to reduce the thickness of the Fe_3_GaTe_2_ layers as much as possible, while keeping the clear magnetic hysteresis and the remnant magnetization at room temperature. Thus, before fabricating Sn‐BSTS/Fe_3_GaTe_2_ heterostructures, we investigated the thickness dependent FM properties by monitoring their AHE. The field‐dependent Hall resistance *R_yx_* (*H*) for atomically‐thin Fe_3_GaTe_2_ flakes display clear hysteresis loops as representatively shown for 7L (**Figure** **2**b; Supplementary Figure S6, Supporting Information), distinct from that of bulk crystals (Figure 2a). A square‐shaped hysteresis and negligible linear field dependence above the coercive field *H*_c_ in *R_yx_* (*H*) are similar with the previous studies on both Fe_3_GaTe_2_ and Fe_3_GeTe_2_^[^ ^50^, ^51^ ^]^ flakes. Upon increasing temperature, the AHE gradually decreases and eventually disappears at *T*_c_ ≈340 K^[^ ^50^ ^]^ for 15 L. Down to thickness of 7 L, *T*_c_ of Fe_3_GaTe_2_ flakes remain above 300 K, whereas the coercive field *H*_c_ exhibits much drastic thickness dependence at room temperature. In bulk, *H*_c_ is almost negligible due to domain formation but enhanced up to ≈300 Oe for 15L, followed by a sudden drop to nearly zero for ≈7 L. These results suggest that for SOT operation with a sizable remnant magnetization, the coercive field *H*_c_, rather than *T*_c_ is the limiting factor at room temperature (Figure 2c, d, e).

**Figure 3:**
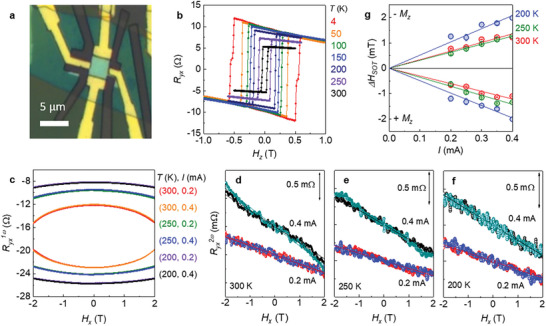
Current induced SOT effective fields from second harmonic measurements. a) Optical images of Sn‐BSTS(32 nm)/Fe_3_GaTe_2_(11 L) heterostructure device. b) Magnetic‐field‐dependent Hall resistance *R_yx_* (*H*) at various temperatures for the heterostructure device. c) The first harmonic Hall resistance (*R_yx_* ^1ω^) as function of the in‐plane magnetic field (*H_x_*) parallel to current direction for various current levels and temperatures. d,e,f) The corresponding second harmonic resistances *R_yx_* ^2ω^(*H_x_*) of Sn‐BSTS/Fe_3_GaTe_2_ device for 300 K (d), 250 K (e) and 200 K (f) after subtracting out the Nernst contribution. The color codes are red (black) and blue (dark green) for The out‐of‐plane magnetization (*M_z_*)> 0 and *M_z_* <0 for *I* = 0.2 mA (0.4 mA). For clarity, the data taken at different currents are vertically shifted. g) Current‐dependent SOT effective field Δ*H*_SOT_ of Sn‐BSTS/Fe_3_GaTe_2_ devices for *M_z_* >0 and *M_z_* <0 at different temperatures. The solid lines represent the linear fits to the data.

**Figure 4:**
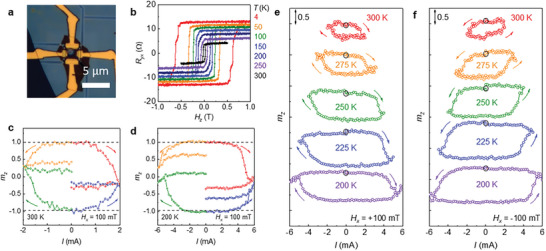
Current induced magnetization switching in Sn‐BSTS/Fe_3_GaTe_2_ devices. a) Optical image of Sn‐BSTS(10 nm)/Fe_3_GaTe_2_(8 L) heterostructure device. b) Magnetic‐field‐dependent Hall resistance *R_yx_* (*H*) at various temperatures for the heterostructure device. c,d) Current‐driven magnetization switching diagram probed by Hall resistance *R_yx_* (*I*) at 300 K (c) and 200 K (d) of Sn‐BSTS/Fe_3_GaTe_2_ device under in‐plane magnetic field *H_x_* = 100 mT. Distinct magnetization switching behaviors are observed depending on the initial magnetization states and the relative direction of the applied current (*I*) and in‐plane magnetic field (*H_x_*). The out‐of‐plane magnetization *m*_z_ is determined by the ratio of *R*_yx_(*I*) against the *R*_yx_ values for full saturation of magnetization. e,f) Temperature dependent current pulse sweeps for Sn‐BSTS/Fe_3_GaTe_2_ device under positive (e) and negative (f) in‐plane field *H_x_*.

Based on the results for individual Sn‐BSTS and Fe_3_GaTe_2_ flakes, we fabricated Sn‐BSTS/ Fe_3_GaTe_2_ heterostructure devices using Sn‐BSTS flakes with a thickness less than 10–40 nm and Fe_3_GaTe_2_ flakes with a thickness ≈10 L. Once exfoliated, these Sn‐BSTS and Fe_3_GaTe_2_ flakes were flipped and stacked using a dry transfer method in an inert atmosphere (See Experimental Section). Based on the temperature dependent sheet resistance *R*_sheet_ (*T*) of each Sn‐BSTS and Fe_3_GaTe_2_ flakes with similar thickness, the parallel conduction model yields the current through the TI layers to be ≈20% of the total current (Figure 1e, g). These observations demonstrate that the contribution of the TSS to the total current can be significant using atomically‐thin bulk‐insulating Sn‐BSTS flakes and ferromagnetic Fe_3_GaTe_2_ layers. Below we focused on two heterostructure devices, Sn‐BSTS(32 nm)/Fe_3_GaTe_2_(11 L) (**Figure** **3**a) and Sn‐BSTS(16 nm)/Fe_3_GaTe_2_(8L) (**Figure** **4**a). Clear field‐dependent hysteresis of *R_yx_* (*H*) with a nearly complete square shape confirms the full remnant magnetization in both heterostructure devices at room temperature (Figure 3b and 4b).

Having established the internal current distribution and FM characteristics for the Sn‐BSTS/Fe_3_GaTe_2_ heterostructures, we proceed to determine the SOT efficiency by employing the second harmonic Hall method. For the second harmonic measurements, an alternating current is applied to the device to generate an oscillating effective SOT field Δ*H*_SOT_, which modulates magnetization about its equilibrium state. This modulation of magnetization introduces a second harmonic term into the Hall voltage, which is asymmetric to the in‐plane magnetic fields (*H_x_*).^[^ ^52^, ^53^ ^]^ In addition, a vertical temperature gradient of the device generates an ordinary Nernst effect signal contributing to the asymmetric and linear in‐plane field dependence of the second harmonic Hall voltage voltage.^[^ ^54^, ^55^ ^]^ Thus, the primary (*R_1_*_ω_) and second (*R*_2ω_) harmonic Hall voltages can be expressed as following equations.

(1) $$R_{1\omega }=\;\pm \frac{1}{2}\Delta R_{A}\left[1-\frac{1}{2}{\left(\frac{H_{x}}{H_{k}}\right)}^{2}\right]$$

(2) $$R_{2\omega }=\;\pm \frac{1}{4}\Delta R_{A}\Delta H_{\mathrm{SOT}}\frac{H_{x}}{H_{k}^{2}}\pm \alpha_{\mathrm{ONE}}H_{x}$$

where *H*_x_ is in‐plane magnetic field parallel to current direction, Δ*R_A_* is the size of the anomalous Hall resistance, *H*_k_ is effective magnetic anisotropy field, α_ONE_ is ordinary Nernst effect coefficient, and Δ*H*_SOT_ is effective SOT field. The SOT contribution can be extracted by subtracting out the ordinary Nernst contribution from the linear fit of *R*_2ω_(*H*_x_) above the saturation magnetic field (Supplementary Figure S7, Supporting Information). Then the effective SOT field is estimated by:

(3) $$\Delta H_{\mathrm{SOT}}=\;-2\left(\frac{\partial V_{2\omega }}{\partial H_{x}}\right)/\left(\frac{\partial^{2}V_{1\omega }}{\partial H_{x}^{2}}\right)$$

that is proportional to the applied currents. Because this second harmonic Hall method requires lower current density than current induced switching, so relatively free from artifacts such as Joule heating,^[^ ^52^, ^56^, ^57^ ^]^ it has been widely employed to determine the SOT efficiency in various SOT devices.^[^ ^18^, ^20^, ^24^ ^]^

The harmonic Hall resistances under in‐plane magnetic fields *H_x_* taken at different temperatures are presented in Figure 3c–f for the of Sn‐BSTS(32 nm)/Fe_3_GaTe_2_(11 L) heterostructure. The parabolic field dependent *R_yx_^1^* ^ω^(*H_x_*) and linear field dependent *R_yx_* ^2ω^(*H_x_*) at low magnetic fields are clearly observed. The effective SOT contribution in *R_yx_* ^2ω^(*H_x_*), obtained after subtracting the signal from the ordinary Nernst effect (Supplementary Figure S7, Supporting Information), exhibits a steep slope with increasing applied current for both positive (*M*_z_ >0) and negative (*M*_z_ <0) magnetization, consistent with the current‐induced SOT. The damping‐like SOT effective field Δ*H*_SOT_ as a function of the applied current is estimated using Equation 3 at different temperatures, as shown in Figure 3g. The average linear slopes obtained for *M*_z_ >0 and *M*_z_ <0 are 3.2  ±  0.12 T/A at room temperature, which increases up to 5.2  ±  0.25 T/A with lowering temperature to 200 K, indicating that the SOT efficiency is enhanced at low temperatures. A similar enhancement of the SOT efficiency with lowering temperature has been observed in the previous BST/CrBST heterostructure devices at low temperature,^[^ ^37^ ^]^ which has been attributed that mixing of spin texture at the TSS due to phonon or magnon scattering is suppressed at low temperatures. This behavior contrasts to the opposite temperature dependence of the SOT efficiency, found in Bi_1‐x_Sb_x_/CoFeB devices where the SOT is dominantly produced by the spin Hall effect of thermally‐excited Dirac electron in the bulk states.^[^ ^58^ ^]^ These results are consistent with the TSS dominant SOT operation in our Sn‐BSTS/Fe_3_GaTe_2_ devices.

**Figure 5:**
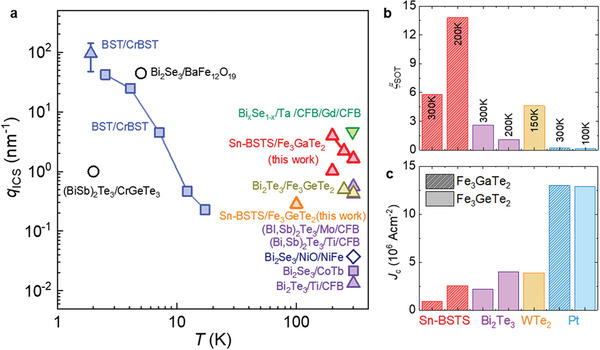
Comparison of SOT efficiencies. a) 2D charge‐to‐spin conversion rate *q*_ICS_ of (Bi,Sb)_2_ *Ch*_3_‐type TI based devices as a function of the measured temperature. Different methods have been employed to determine *q*_ICS_, including the second harmonic measurements at high magnetic fields (lower triangles) and low magnetic fields (upper triangles), the magnetic hysteresis shift (square), current‐induced magnetization switching (circles) and the spin transfer ferromagnetic resonance (diamonds). b–c) The SOT efficiency *ξ*_SOT_ (b) and the magnetization switching current density *J*_c_ (c) for the SOT devices with FM layers of Fe_3_GaTe_2_ (hatched) or Fe_3_GeTe_2_ (shaded), together with the Sn‐BSTS (red), Bi_2_Te_3_ (purple), WTe_2_ (orange) and Pt (cyan) layers.

To compare the SOT efficiency (*ξ*_SOT_) of our devices in the previous studies, we estimate it using the expression $\xi_{\mathrm{SOT}}=\frac{2e}{\hbar }\times \;M_{s}t_{\mathrm{FM}}\times \frac{\mu_{0}\Delta H_{\mathrm{SOT}}}{J_{3D}}$, where *e* is electron charge, *ħ* is the reduced Planck constant, *t*_FM_ is the thickness of FM layers, *M*_s_ is the saturation magnetization, and *J*_3D_ is the current density in the TI layers. Considering the fact that the TSS conduction dominates in our bulk‐insulating TI layers (Figure 1f) and the bottom TSS is active for SOT to the Fe_3_GaTe_2_ layers, we estimate *J*_3D_ using effective thickness of the TSS, *t*_TSS_ ≈3.5 nm, which have been extracted from the film thickness below which the top and bottom surfaces become hybridized.^[^ ^59^, ^60^, ^61^, ^62^ ^]^ With the bulk saturated magnetization of Fe_3_GaTe_2_ (Supplementary Figure S3, Supporting Information), the damping‐like SOT efficiencies are determined to be *ξ*_SOT_ ≈5.77 at 300 K and enhanced up to *ξ*_SOT_ ≈13.8 at 200 K for Sn‐BSTS/Fe_3_GaTe_2_ devices. Using the 2D current density $j_{2D}=J_{3D}\;\times \frac{t_{\mathrm{TI}}}{t_{\mathrm{TSS}}}$,^[^ ^39^ ^]^ we also estimate the interfacial charge‐to‐spin conversion rate (*q*_ICS_) to be *q*_ICS_ ≈1.65 nm^−1^ at room temperature, which is again enhanced up to *q*_ICS_ ≈3.95 nm^−1^ at 200 K (**Figure** **5**a). These parameters *ξ*_SOT_ and *q*_ICS_ for SOT efficiency are an order of magnitude larger than those of Pt/Fe_3_GeTe_2_ and Pt/Fe_3_GaTe_2_ devices,^[^ ^23^, ^24^, ^25^, ^26^ ^]^ confirming the efficient SOT from the TSS of the TI.

Current‐induced magnetization switching by efficient SOT is clearly observed in Sn‐BSTS(16 nm)/Fe_3_GaTe_2_(8 L) heterostructures (Figure 4). For the switching experiments, we applied 10 ms‐long current pulses with different magnitudes. Following this, reading pulses of 20 µA were utilized to monitor the magnetization of Fe_3_GaTe_2_ layer via the AHE at different temperatures. For deterministic switching, a constant in‐plane field of *H_x_* = 100 mT was applied during the experiments. By reversing directions of either the pulsed current or the initial magnetization, we clearly show that for the *M*_z_ > 0 (*M*_z_ <0) initial state, only +*I* (−*I*) current induces switching at *H_x_ >* 0 field (Figure 4c,d). The values of critical switching current, *I*_c_, are ≈1.5 mA at 300 K and ≈5 mA at 200 K. By comparing the anomalous Hall resistance for the fully saturated magnetization states, we extract the current‐induced change in the normalized magnetization (Δ*m*_z_) is ≈0.5 at 300 K, smaller than the ideal value of Δ*m*_z_ = 2, indicating that magnetization switching is partly achieved, presumably due to multiple domain formation as a result of Joule heating^[^ ^63^, ^64^ ^]^ or the spreading of in‐plane current distribution within the Hall bar device^[^ ^65^, ^66^ ^]^ Upon lower temperature, the current‐induced hysteresis become stronger with enhanced Δ*m*_z_ up to ≈1 at 200 K. The clockwise and counter‐clockwise current‐induced magnetization hysteresis curves are consistently observed with *H_x_* > 0 and *H_x_* <0, respectively, for Sn‐BSTS/Fe_3_GaTe_2_ device (Figure 4e,f). These results suggest that, in line with the second harmonic measurements (Figure 3), efficient SOT operation can be achieved with the atomically‐thin Fe_3_GaTe_2_ layers in Sn‐BSTS/Fe_3_GaTe_2_ heterostructures.

Given that the current distributions between the Fe_3_GaTe_2_ and Sn‐BSTS layers, as well as within the Sn‐BSTS layers, are significantly temperature‐dependent, the possible Joule heating effect at high current influencing the SOT performance needs consideration. We evaluate the electronic temperature (*T*_e_) by monitoring the anomalous Hall signal *R_yx_^A^* during the application of the current pulse. By comparing the *R_yx_^A^* (*H* = 0) value, measured at a small current of 20 µA, *R_yx_^A^* (*H* = 0) at higher current up to ≈2 mA is reduce to ≈80%, indicating that *T*_e_ at a critical switching current remains well below *T*_c_. Taking into account the internal current distribution in the Sn‐BSTS layers, the values of switching current density, *J*_c_, flowing through the bottom TSS are calculated to be ≈0.91×10^6^ A cm^−2^ at 300 K and ≈2.56×10^6^ A cm^−2^ at 200 K, which are an order of magnitude smaller than *J*_c_ of Pt/Fe_3_GaTe_2_ devices^[^ ^25^, ^26^ ^]^ and comparable with other TI‐based SOT devices.^[^ ^16^, ^17^, ^18^, ^19^, ^20^, ^67^, ^68^, ^69^ ^]^ These observations confirm efficient SOT switching in vdW heterostructures of Sn‐BSTS and Fe_3_GaTe_2_ layers.

## 3 Discussion

We now compare the SOT efficiency of the previously studied SOT devices with those of our vdW SOT devices shown in Figures 3 and 4 as well as shown in Supplementary Figures S9–S11 (Supporting Information). First, we focus on the interfacial charge‐to‐spin conversion efficiency *q*_ICS_ using the TSS of (Bi,Sb)_2_ *Ch*_3_‐type TI layers (Figure 5a). As discussed above, when utilizing bulk‐insulating, and thus low‐conducting TI layers for SOT device, the current shunting problem is one of the limiting factors for efficient SOT operation. One approach to mitigate this problem is inserting a magnetic insulating layers such as antiferromagnetic insulating NiO layers, between TI (Bi_2_Se_3_) and FM (NiFe) layers to block current leakage.^[^ ^31^, ^70^ ^]^ In such a device configuration, magnon‐mediated spin transfer across NiO layers has found to exhibit weak interfacial charge‐to‐spin conversion with *q*_ICS_ ≈0.037 nm^−1^. Using FM insulating (FMI) layers is another approach, as exemplified by Cr‐doped (Bi,Sb)_2_Te_3_/(Bi,Sb)_2_Te_3_,^[^ ^18^, ^37^, ^71^, ^72^ ^]^ Bi_2_Se_3_/BaFe_12_O_19_^[^ ^73^ ^]^ and (Bi,Sb)_2_Te_3_/CrGeTe_3_^[^ ^41^ ^]^ heterostructures. While spin can be efficiently transferred to the FMI, resulting in high efficiency *q*_ICS_ ≈140–425 nm^−1^ for Cr‐(Bi,Sb)_2_Te_3_/(Bi,Sb)_2_Te_3_,^[^ ^18^ ^]^ *q*_ICS_ ≈300 nm^−1^ for Bi_2_Se_3_/BaFe_12_O_19_,^[^ ^73^ ^]^ and *q*_ICS_ ≈6 nm^−1^ for (Bi,Sb)_2_Te_3_/CrGeTe_3_.^[^ ^41^ ^]^ However, this efficient SOT operation is limited only at low temperatures below 20 K due to the low *T*_c_ of the FMI layers. At room temperature, except the one with granular Bi_1‐_ *_x_* Se*_x_* films with *q*_ICS_ ≈4.5 nm^−1^ due to quantum confinement,^[^ ^19^ ^]^ the SOT devices with (Bi,Sb)_2_ *Ch*_3_‐type TI layers and FM metallic layers show *q*_ICS_ ≈0.01–0.6 nm^−1^. In our Sn‐BSTS/Fe_3_GaTe_2_ devices, we achieved a TSS conduction contribution up to ≈20% and a large charge‐to‐spin conversion with *q*_ICS_ ≈1.65 nm^−1^, which is larger than those observed in typical TI/FM devices at room temperatures implying the validity of our approach.

Among other SOT devices using HMs, TIs, or topological semimetals as SOT layers, together with FM vdW ferromagnets Fe_3_GeTe_2_ and Fe_3_GaTe_2_, our vdW heterostructure Sn‐BSTS/Fe_3_GaTe_2_ devices show enhanced SOT efficiency *ξ*_SOT_ and reduced switching current density *J*_c_ as shown in Figure 5b,c. Comparing with the Pt/Fe_3_(Ga,Ge)Te_2_ devices with *ξ*_SOT_ ≈0.12 – 0.22,^[^ ^23^, ^24^, ^25^, ^26^ ^]^ we found that the SOT efficiency is one order of magnitude higher in Sn‐BSTS/Fe_3_GaTe_2_ devices with *ξ*_SOT_ ≈5.77–13.8. Consistently, the values of switching current density *J*_c_ of Sn‐BSTS/Fe_3_GaTe_2_ (≈0.91 × 10^6^ A cm^−2^) are much smaller than that of *J*_c_ of Pt/Fe_3_GeTe_2_ and Pt/Fe_3_GaTe_2_ (≈13 × 10^6^ A cm^−2^). This enhanced SOT performance with a high *ξ*_SOT_ and a low *J*_c_ in our Sn‐BSTS/Fe_3_GaTe_2_ devices at room temperature are much better than the relatively low *J*_c_ ≈5.8 × 10^6^ A cm^−2^ at 200 K in a MBE‐grown (Bi,Sn)_2_Te_3_/Fe_3_GeTe_2_ device,^[^ ^74^ ^]^ and *J*_c_ ≈3.9 × 10^6^ A cm^−2^ with *ξ*_SOT_ ≈4.6 found at 150 K in a vdW heterostructure WTe_2_/Fe_3_GeTe_2_ devices.^[^ ^28^ ^]^ Moreover, in the case of Sn‐BSTS/Fe_3_GeTe_2_ devices (Supplementary Figure S12, Supporting Information), we found that as the thickness of Fe_3_GeTe_2_ layers is reduced from 7L to 3L, the switching current decreases and the portion of current‐induced magnetization change Δ*m*_z_ systematically increases. Therefore, thickness control of the FM and TI layers and their vdW stacking is essential to realize highly efficient SOT operation at room temperature.

In a recent study on MBE‐grown Bi_2_Te_3_/Fe_3_GeTe_2_ vdW heterostructure devices,^[^ ^30^ ^]^ a high efficiency of *ξ*_SOT_ ≈0.6–2.6 and a low switching current *J*_c_ ≈2.2 × 10^6^A cm^−2^ was claimed at room temperature, emphasizing the critical role of the TSS and clean vdW interface. These SOT performance falls short when compared to our device's with *ξ*_SOT_ ≈5.77 and *J*_c_ ≈0.91 × 10^6^A cm^−2^ at 300 K. Furthermore, the observed current‐induced magnetization switching Δ*m*_z_ is markedly small, ≈4% at room temperature, an order of magnitude smaller than Δ*m*_z_ ≈25% found in our devices (Figure 4e,f). This difference can be attributed to the weak interfacial ferromagnetism induced by the proximity effect at 300 K, beyond the intrinsic *T*_c_ ≈220 K of Fe_3_GeTe_2_ in the MBE‐grown Bi_2_Te_3_/Fe_3_GeTe_2_ heterostructures. This comparison emphasizes that, for improved SOT operation at room temperature, importance extends not only to the TI layers but also to the FM layers possessing intrinsic room‐temperature ferromagnetism with strong PMA.

There is plenty of room for further optimizing SOT performance in our device scheme. Several other vdW materials exhibit intrinsic ferromagnetism at room temperature, including Fe_5_GeTe_2_,^[^ ^75^, ^76^ ^]^ MnSiTe_3_, and MnGeTe_3_,^[^ ^77^ ^]^ which can be used for the FM layers with a larger remnant magnetization and strong PMA in the vdW SOT devices. We also observed that the coercive field *H*_c_ of Fe_3_GaTe_2_ layers becomes larger in Sn‐BSTS/Fe_3_GaTe_2_ heterostructures than in Fe_3_GaTe_2_ flakes alone (Supplementary Figure S6, Supporting Information). This suggests that with the aid of proximity‐induced PMA, we can further reduce the thickness Fe_3_GaTe_2_ flakes, thereby decreasing the conductivity of the FM layers^[^ ^44^ ^]^ and the total magnetization per area of FM layers, *M*_s_ *t*_FM_, favouring a decrease of the switching current. Moreover, in an odd‐number‐layer thick Fe_3_(Ga,Ge)Te_2_, the inversion symmetry is broken, introducing current‐induced SOT within the Fe_3_(Ga,Ge)Te_2_ layer.^[^ ^78^, ^79^, ^80^ ^]^ This means that the leakage current through the Fe_3_(Ga,Ge)Te_2_ layers can assist in magnetization switching and enhancing the SOT efficiency. Last but not least, the proximity effect between the TI and the FM layers modulates the Fermi energy or the helical spin texture of the TSS in the TI layers, as well as the magnetic properties of the FM layers.^[^ ^34^, ^81^, ^82^, ^83^ ^]^ This mutual proximity effect can further optimize the SOT performance. We envisioned that these unique approaches for vdW heterostructures, combined with their topological nature, hold the promise of highly‐efficient SOT devices, representing a significant advancement toward realizing all‐vdW‐materials‐based spintronics.

## 4 Experimental Section

### Single‐Crystal Growth

Single crystals of Sn doped Bi_1.1_Sb_0.9_Te_2_S were grown by a vertical Bridgman technique (VBT). A mixture of Sn, Bi, Sb, Te, and S with 5N high purity, based on the chemical formula of Sn_0.02_Bi_1.08_Sb_0.9_Te_2_S, was placed in a sealed quartz ampoule. The ampule was heated to 900 °C within 10 h, then cooled to 750 °C at a rate of 10 °C h^−1^. The annealing process was done at 750 °C for 150 h, after which the furnace was cooled down to 500 °C for 25 h. Single crystals of Fe_3_GaTe_2_ were grown by a chemical vapor transport method with iodine as a transport agent. A mixture of Fe(99.998%), Ga(99.9995%), and Te(99.999%) in a molar ratio of 3:1:2 was placed in an evacuated quartz ampoule that was heated at 750 °C/700 °C with a temperature gradient of 5 °C cm^−1^ for 7 days in a two‐zone furnace. Fe_3_GeTe_2_ single crystals were grown using a similar method with Ge(99.999%) instead of Ga. The crystallinity and stoichiometry of Sn‐BSTS, Fe_3_GaTe_2_ and Fe_3_GeTe_2_ crystals were confirmed by X‐ray diffraction (Supplementary Figure S2, Supporting Information) and Energy dispersive X‐ray spectroscopy.

### Device Fabrications

A Fe_3_GaTe_2_ as well as Fe_3_GeTe_2_ single crystal was exfoliated with Al_2_O_3_‐assisted cleaving method.^[^ ^44^ ^]^ Aluminum oxide layers with a thickness 50–60 nm was evaporated on the Fe_3_GaTe_2_ crystal, which was then transferred on thermal released tape (Nitto Denko corp.). Nano‐flake of Sn‐BSTS was cleaved with the same Al_2_O_3_‐assited cleaving method described above and then flipped using Gelpak. For Sn‐BSTS flakes with a thickness > 30 nm were exfoliated directly onto Gelpak. The Sn‐BSTS flakes are then transferred onto Fe_3_GaTe_2_ flakes. During this process, the stage temperature was gradually increased up to 80 °C to remove any bubbles between Sn‐BSTS and Fe_3_GaTe_2_ layers. All exfoliation and transfer procedures were conducted in an inert argon atmosphere. To prevent degradation of the Sn‐BSTS/Fe_3_GaTe_2_ heterostructures, a layer of aluminum oxide with a thickness of 2.6 nm was deposited using e‐beam evaporation, without exposing the sample to air. The thickness of Fe_3_GaTe_2_ layer was determined by the optical contrast (Figure 1d), and the thickness of Sn‐BSTS layer is measured by atomic force microscopy after the deposition of the aluminum oxide capping layer (Supplementary Figure S5, Supporting Information). Electrodes were patterned using e‐beam lithography, and the electrode contacts to the heterostructures were established through in situ Argon ion milling, followed by metal evaporation of Cr(5 nm)/Au (100 nm). Finally, to ensure the well‐defined current path in the devices, the Sn‐BSTS/Fe_3_GaTe_2_ layers were shaped into a Hall bar geometry using focused ion beam (Ga ion) micro‐machining or in situ Argon ion milling.

### Transport and SOT Measurements

All the electrical measurements were performed in an Oxford 7T horizontal magnet employing an one‐axis rotator probe. A synchronous source measure system (Lakeshore, M81‐SSM) was used to measure the transport characteristics at an alternating current of a frequency 17.777 Hz. For the second harmonic measurements, the in‐plane magnetic field (*H_x_*) was swept, while measuring the first (*R_yx_* ^1ω^) and second (*R_yx_* ^2ω^) harmonic Hall resistance signals simultaneously, after initializing the magnetization under an out‐of‐plane magnetic field. The second harmonic Hall signals were considered that exhibited antisymmetric behavior under in‐plane magnetic field sweep. By subtracting the linear field slope of *R_yx_* ^2ω^(*H_x_*) at high magnetic fields above the coercive field of the Fe_3_GaTe_2_ layers, the low‐field linear slope of the second harmonic signals within the magnetic field range of slope ± 1.5 T was obtained, which is used to determine the SOC efficiency. Magnetization switching experiment was performed with a source and voltage meters (Keithley 6221 and 2182). Initially, a 10 ms‐long write current was applied, followed by a 100 ms waiting period. Subsequently, the Hall resistance was measured using a 10 µA DC pulse. The electronic temperature of the device during the DC pulse measurements was estimated by comparing the size of the anomalous Hall resistance jump while sweeping the out‐of‐plane magnetic field with those taken at different temperatures with a the low current of 10 µA.

## Conflict of Interest

The authors declare no conflict of interest.

## Author Contributions

G.S.C. and S.P. contributed equally to this work. G.‐H.L., W.J.C, and J.S.K conceived and supervised the project. G.S.C., E.‐S.A., J.H.B., and I.S. fabricated the samples. G.S.C., S.P., and E.‐S.A. performed the transport experiments. B.T.K., C.J.W., and S.‐W.C. grew single crystals. G.S.C., E.‐S.A., S.P., G.‐H.L., and J.S.K analyzed the transport data. G.S.C., S.P., and J.S.K co‐wrote the manuscript. All authors discussed the results and commented on the paper.

## Supporting information

Supporting Information

## Data Availability

The data that support the findings of this study are available from the corresponding author upon reasonable request.
