# Supplementary material for: Highly Efficient Room‐Temperature Spin‐Orbit‐Torque Switching in a Van der Waals Heterostructure of Topological Insulator and Ferromagnet
Source: Adv Sci (Weinh). 2024 Mar 22;11(21):2400893. doi: 10.1002/advs.202400893 (PMC11151020; doi:10.1002/advs.202400893)
Supplement: Supplementary file 1 — Supporting Information [file ADVS-11-2400893-s001.pdf]

ADVANCED SCIENCE
Open Access

Supporting Information

for *Adv. Sci.*, DOI 10.1002/advs.202400893

Highly Efficient Room-Temperature Spin-Orbit-Torque Switching in a Van der Waals Heterostructure of Topological Insulator and Ferromagnet

Gyu Seung Choi, Sungyu Park, Eun-Su An, Juhong Bae, Inseob Shin, Beom Tak Kang, Choong Jae Won, Sang-Wook Cheong, Hyun-Woo Lee, Gil-Ho Lee, Won Joon Cho* and Jun Sung Kim**

Supporting Information

Highly Efficient Room-Temperature Spin-Orbit-Torque Switching in a Van der Waals heterostructure of Topological Insulator and Ferromagnet

Gyu Seung Choi[1,2*], Sungyu Park[1*], Eun-Su An[1,2], Juhong Bae[1], Inseob Shin[1], Beom Tak Kang[1,2], Choong Jae Won[4], Sang-Wook Cheong[4,5,6], Hyun-Woo Lee[1], Gil-Ho Lee[1,†], Won Joon Cho[3,†] and Jun Sung Kim[1,2,†]

[1] Department of Physics, Pohang University of Science and Technology, Pohang 37673, Republic of Korea

[2] Center for Artificial Low Dimensional Electronic Systems, Institute for Basic Science (IBS), Pohang 37673, Korea

[3] Device Research Center, Samsung Advanced Institute of Technology (SAIT), Samsung Electronics Co., Ltd, 130 Samsung-ro, Yeongtong-gu, Suwon-si, Gyeonggi-do 16678, Republic of Korea

[4] Max Planck POSTECH/Korea Research Initiative, Center for Complex Phase of Materials, Pohang 37673, Republic of Korea.

[5] Laboratory for Pohang Emergent Materials, Department of Physics, Pohang 37673, Korea

[6] Rutgers Center for Emergent Materials and Department of Physics and Astronomy, Rutgers University, Piscataway, New Jersey 08854, USA

* Equal contribution

† Correspondence and requests for materials should be addressed to G.H.L (lghman@postech.ac.kr), W.J.C (wj7.cho@samsung.com) and J.S.K. (js.kim@postech.ac.kr).

1. Device Fabrication procedure using Aluminum assisted cleaved Fe_3GaTe_2 & Sn-BSTS

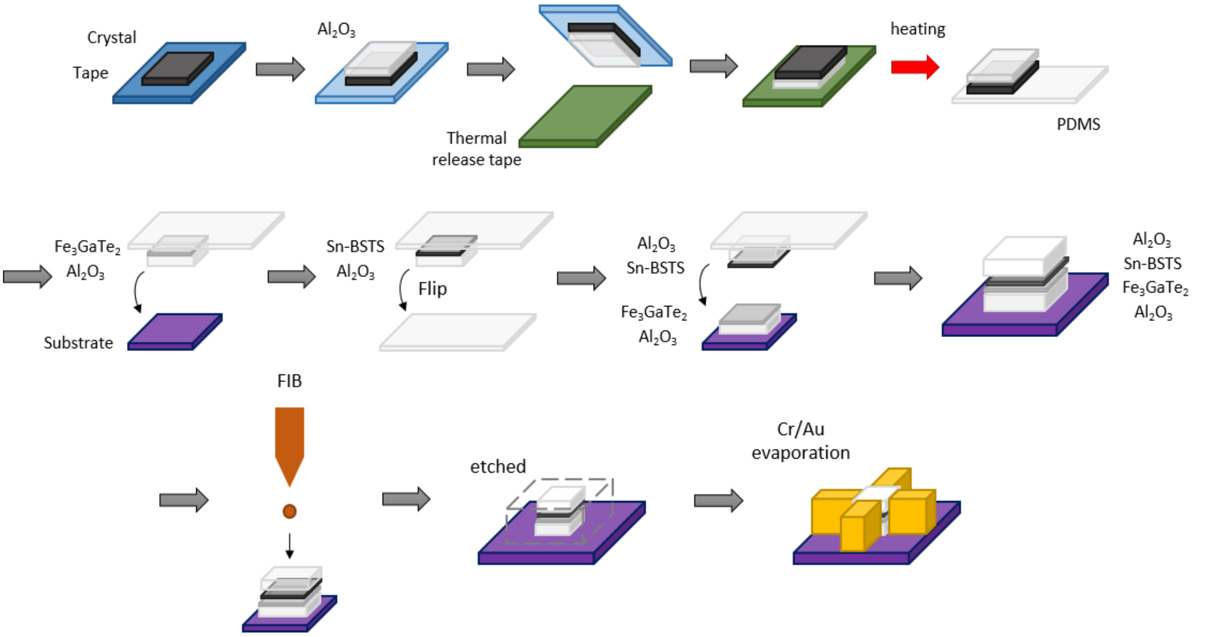

Figure S1. Schematics of fabrication procedures for vdW heterostructure devices. Al_2O_3 deposition onto the surface of a bulk crystal, either Fe_3GaTe_2 or Sn-BSTS is followed by mechanical exfoliation using a thermal release tape and a PDMS. While the Fe_3GaTe_2/Al_2O_3 layers were transferred to the substrate, the Sn-BSTS/ Al_2O_3 layers were flipped using Gelpak and then transferred on top of the Fe_3GaTe_2/Al_2O_3 layers. Focused Ion Beam (FIB)-based micro-machining is employed to shape the heterostructure device, before evaporation and patterning of Cr/Au electrodes.

2. Single crystal images and XRD patterns

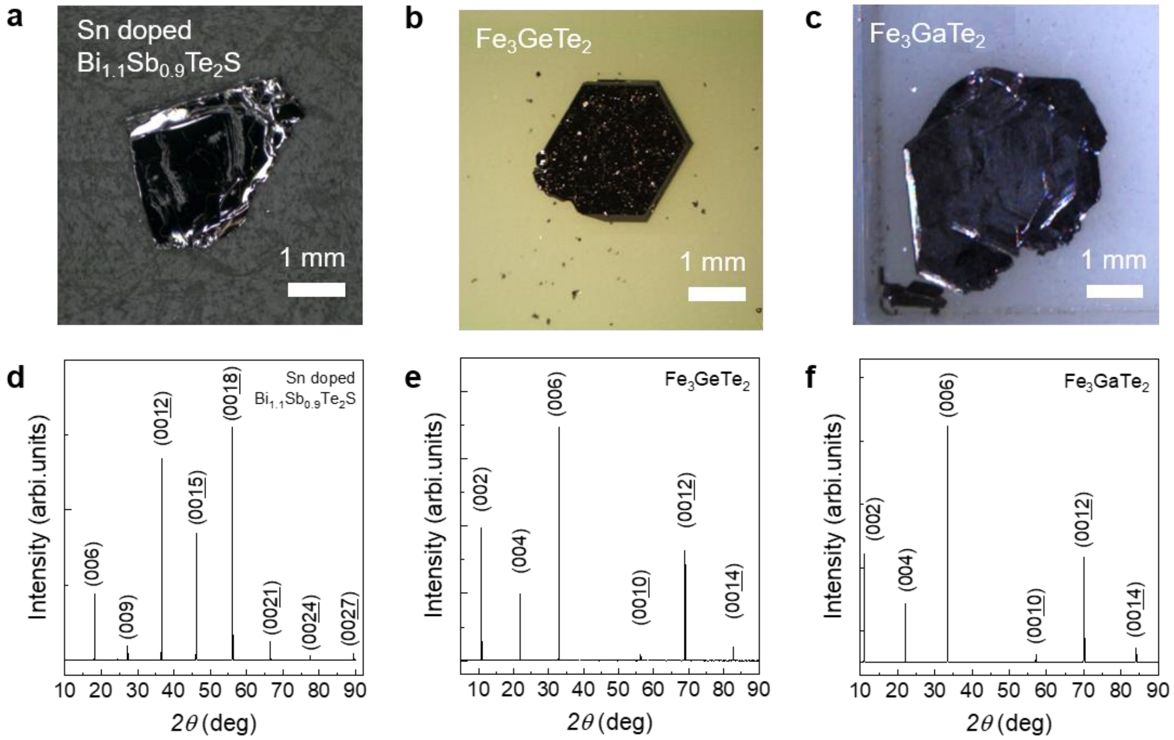

Figure S2. a-c, Optical images for Sn-doped $Bi_{1.1}Sb_{0.9}Te_2S$ (**a**), Fe_3GeTe_2 (**b**) and Fe_3GaTe_2 (**c**) single crystals. **d-f,** X-ray diffraction patterns of single crystals, showing (00*l*) Bragg peaks.

3. Magnetic properties of single crystal, Fe_3GeTe_2 and Fe_3GaTe_2

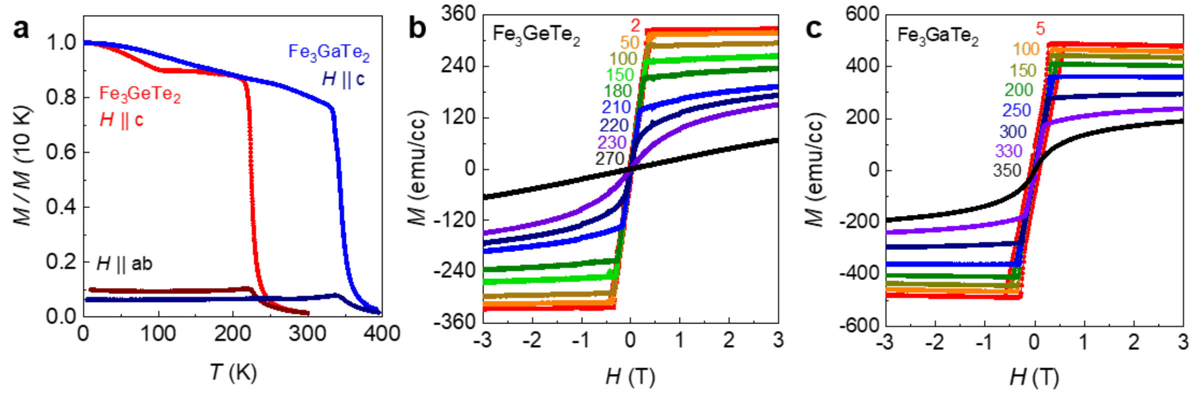

Figure S3. **a,** Temperature-dependent magnetization *M(T)* taken at *H* = 1 kOe for bulk Fe_3GeTe_2 and Fe_3GaTe_2 crystals for the out-of-plane (*H* || *c*) and in-plane (*H* || *ab*) magnetic fields. Typical ferromagnetic phase transition occurs at the critical temperature $T_c \sim 220$ K for Fe_3GeTe_2 and ~ 350 K for Fe_3GaTe_2. **b,c,** Magnetic field dependent magnetization *M(H)* of Fe_3GeTe_2 (b) and Fe_3GaTe_2 (c) taken at various temperatures under out-of-plane magnetic fields (*H* || *c*).

4. Temperature dependent sheet resistance of Fe_3GaTe_2 and Sn-BSTS and TSS current ratio in heterostructure

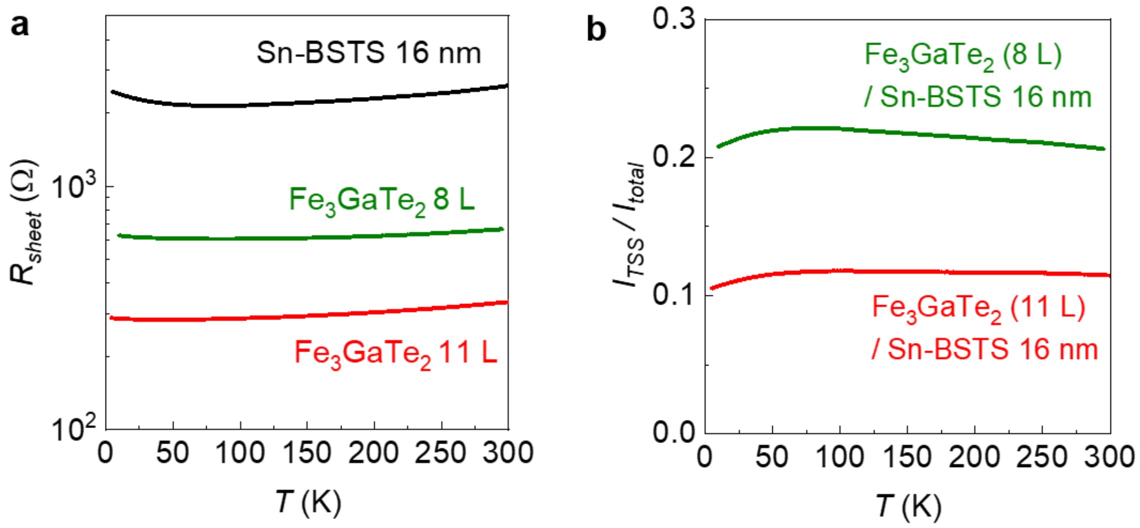

Figure S4. **a,** Temperature-dependent sheet resistance $R_{sheet}(T)$ for 16 nm-thick Sn-BSTS flake and 8 L- and 11 L-thick Fe_3GaTe_2 flakes. **b,** The relative contribution of the TSS to the total current as a function of temperature.

5. Optical images and thickness information for Fe_3GaTe_2 and Sn-BSTS composing of heterostructure

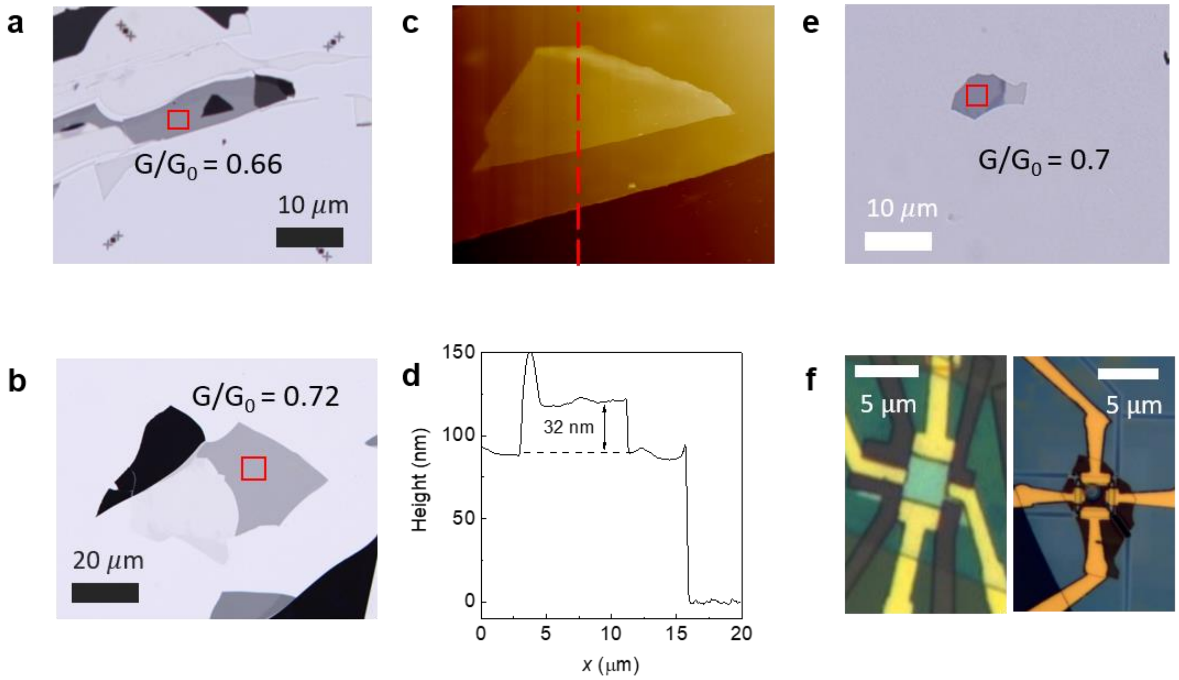

Figure S5. a,b, Transmitted optical images of thin flakes of Fe_3GaTe_2 11 L (**a**) and 8 L (**b**). **c, d,** Atomic force microscopy (AFM) image (**c**) of Sn-BSTS (32 nm)/Fe_3GaTe_2 (11 L) and corresponding height profile (**d**). **e**, Transmitted optical image of thin Sn-BSTS flake in Sn-BSTS (10 nm)/Fe_3GaTe_2 (8 L) heterostructure device. **f**, Optical images Sn-BSTS/Fe_3GaTe_2 devices.

6. magnetic properties of Fe3GaTe2 devices

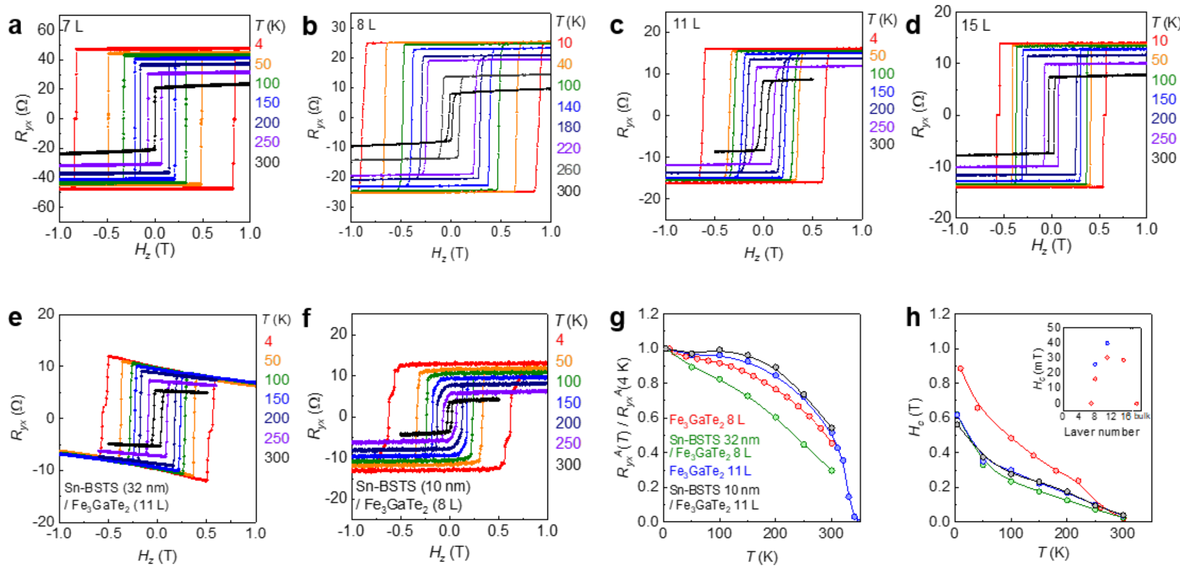

Figure S6. a-f, Magnetic-field-dependent Hall resistance $R_{yx}(H)$ at various temperatures for Fe_3GaTe_2 7 L (**a**), 8 L (**b**), 11 L (**c**), 15 L (**d**) and Sn-BSTS (32 nm)/Fe_3GaTe_2 (11 L) (**e**) and Sn-BSTS (10nm)/Fe_3GaTe_2 (8 L) (**f**). **g, h,** Temperature-dependent anomalous Hall resistance $R_{yx}^{A}(T)$ (**g**) at zero magnetic field, normalized by $R_{yx}^{A}(T\!=\!4\text{ K})$ and coercivity field H_c(T) (**h**) for Fe_3GaTe_2 8 L and 11 L and Sn-BSTS (32 nm)/Fe_3GaTe_2 (11 L) and Sn-BSTS (10 nm)/Fe_3GaTe_2 (8 L) devices. The inset of **h** shows thickness-dependent coercivity field H_c at room temperature (300 K) for individual Fe_3GaTe_2 flakes (red) and for Sn-BSTS/Fe_3GaTe_2 heterostructures (blue).

7. Second harmonic measurement results for Sn-BSTS/Fe_3GaTe_2 device

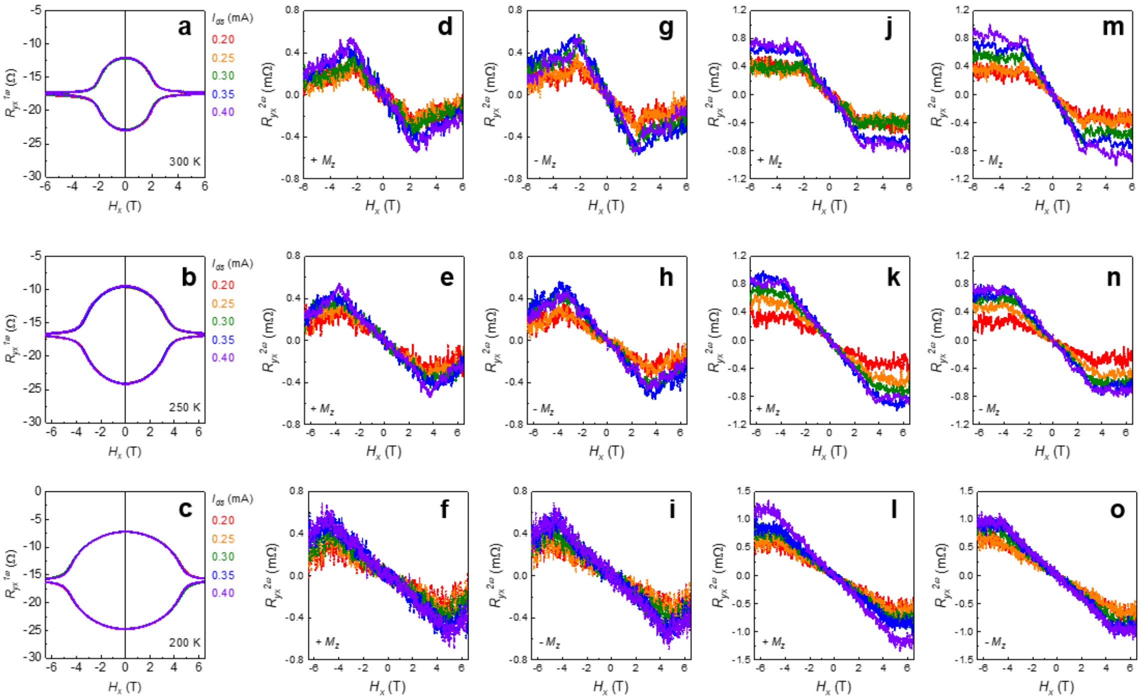

Figure S7. a-c, The first harmonic Hall resistance ($R_{yx}^{1\omega}$) as a function of the in-plane magnetic field (H_x) parallel to the current direction at various current levels for Sn-BSTS (32 nm)/Fe_3GaTe_2 (11L) device, measured at 300 K (**a**), 250 K (**b**) and 200 K (**c**). **d-i,** The corresponding second harmonic resistances $R_{yx}^{2\omega}(H_x)$ for $M_z > 0$ and $M_z < 0$ measured at 300 K (**d, g**), 250 K (**e, h**) and 200 K (**f, i**). **j-o,** The second harmonic resistances $R_{yx}^{2\omega}(H_x)$ after subtracting the Nernst contribution at each current level for $M_z > 0$ and $M_z < 0$ measured at 300 K (**j, m**), 250 K (**k, n**) and 200 K (**l, o**). The same color codes are applied as in **a, b, c.**

8. Anomalous Hall signal with different applied currents for Sn-BSTS/Fe3GaTe2 device

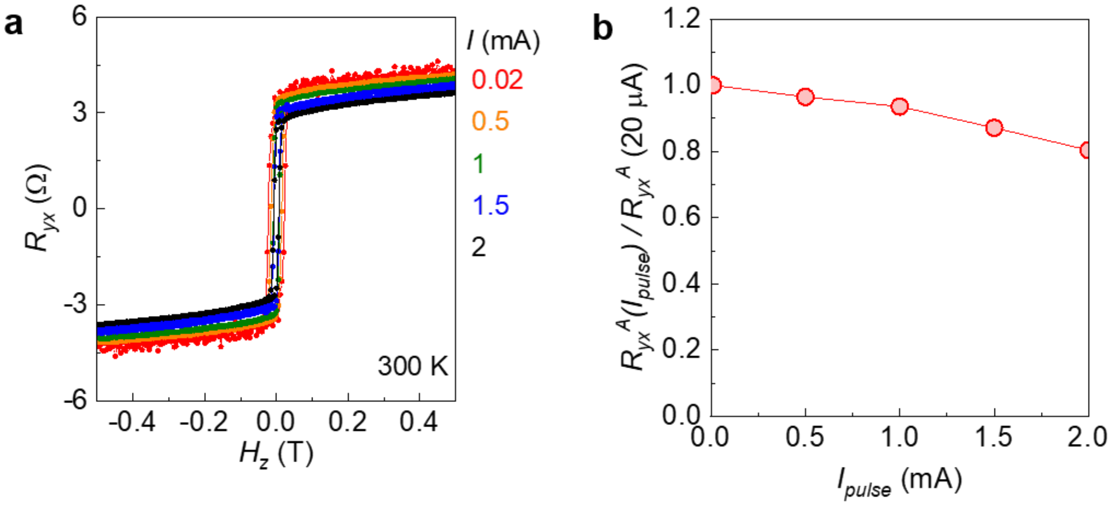

Figure S8. **a,** Magnetic-field-dependent Hall resistance $R_{yx}(H_z)$ at various DC pulse level for Sn-BSTS(10 nm)/Fe_3GaTe_2(8 L) device measured at 300 K. **b,** Anomalous Hall resistance $R_{yx}^{A}(I_{pulse})$ at zero magnetic field, normalized by R_{yx}^{A} (I_{pulse}=20 μA) as a function of the applied current in the Sn-BSTS/Fe_3GaTe_2 device.

9. Temperature dependent sheet resistance and magnetic properties of additional Sn-BSTS/Fe3GeTe2 and Sn-BSTS/Fe3GaTe2 devices

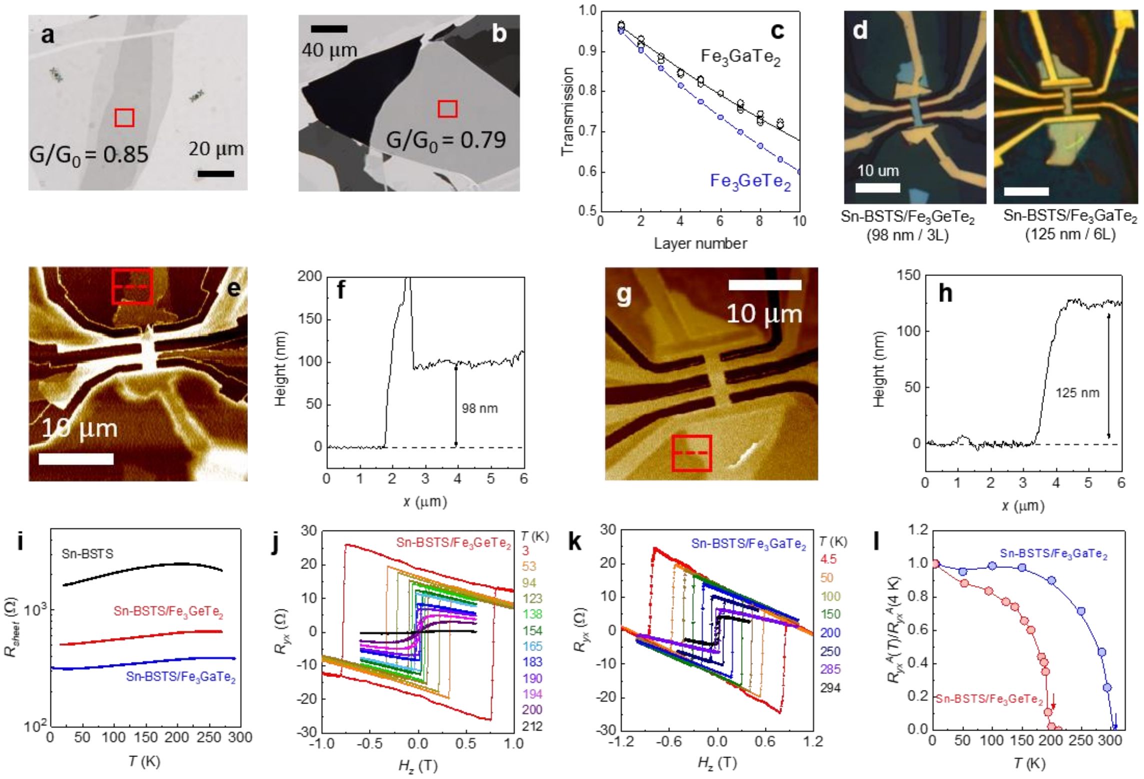

Figure S9. a,b, Optical images in the transmission mode of Fe_3GeTe_2 **(a)** and Fe_3GaTe_2 **(b)** for the Sn-BSTS/FGT devices. The thickness of Fe_3GeTe_2 and Fe_3GaTe_2 flakes is estimated to be 3 layers and 6 layers from the optical transmission curve. **c,** Layer-dependent optical transmission G normalized by that of the substrate (G_0) for Fe_3GeTe_2 (Blue) and Fe_3GaTe_2 (Black) nanoflakes. The solid lines represent the best fit to the Beer-Lambert law. **d,** Optical images of Sn-BSTS/Fe_3GeTe_2 and Sn-BSTS/Fe_3GaTe_2 devices. **e-h,** Atomic force microscopy (AFM) images Sn-BSTS/Fe_3GeTe_2 **(e)** and Sn-BSTS/Fe_3GaTe_2 devices **(g)**. Height profiles for Sn-BSTS/Fe_3GeTe_2 (f) and Sn-BSTS/Fe_3GaTe_2 (**h**) devices, taken along the dashed lines shown in **e** and **h**, respectively. **i**, Temperature-dependent sheet resistance $R(T)$ of 82 nm-thick Sn-BSTS flake, Sn-BSTS(98 nm)/Fe_3GeTe_2(3 layers) and Sn-BSTS(125 nm)/Fe_3GaTe_2(6 layers) heterostructure devices. **j,k,** Magnetic-field-dependent Hall resistance $R_{yx}(H)$ at various temperatures for Sn-BSTS/Fe_3GeTe_2 **(j)** and Sn-BSTS/Fe_3GaTe_2 **(k)** devices. **l**, Temperature-dependent anomalous Hall resistance $R_{yx}^{A}(T)$ at zero magnetic field, normalized by $R_{yx}^{A}(T=4\ K)$, for both Sn-BSTS/FGT devices. The critical temperature T_c at which $R_{yx}^{A}(T)/R_{yx}^{A}(T=4\ K)$ drops to zero is indicated by the arrows.

10. Second harmonic measurements for additional Sn-BSTS/Fe_3GeTe_2 and Sn-BSTS/Fe_3GaTe_2 devices

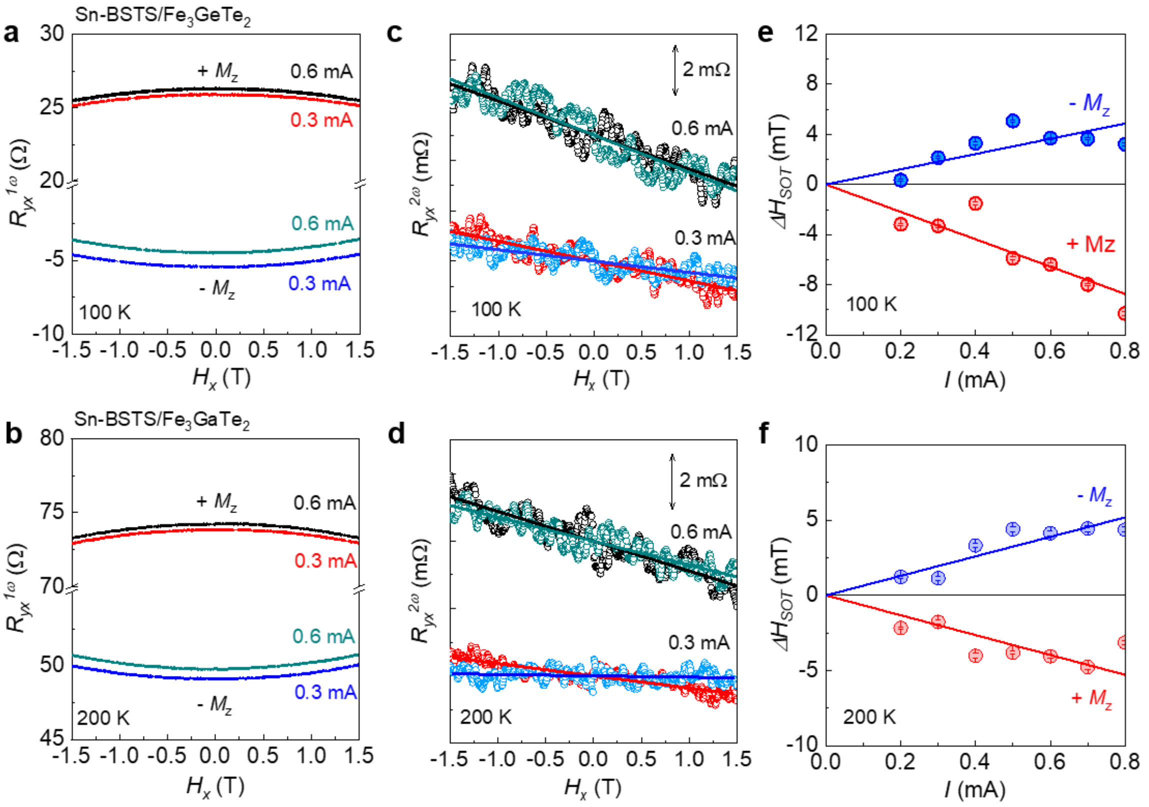

Figure S10 | Current induced SOT effective fields from second harmonic measurements. a,b, The first harmonic Hall resistance ($R_{yx}^{1\omega}$) as function of the in-plane magnetic field (H_x) parallel to current direction for (**a**) Sn-BSTS/Fe_3GeTe_2 and (**b**) Sn-BSTS/Fe_3GaTe_2 devices. The representative $R_{yx}^{1\omega}$ (H_x) curves with a current I = 0.3 mA and 0.6 mA are shown for both M_z > 0 and M_z <0. **c,d,** The corresponding second harmonic resistances $R_{yx}^{2\omega}(H_x)$ for (**c**) Sn-BSTS/Fe_3GeTe_2 and (**d**) Sn-BSTS/Fe_3GaTe_2 devices, after subtracting out the Nernst contribution. The same color codes are applied as in **a** and **b**, and for clarity, the data taken at different currents I = 0.3 mA and 0.6 mA are vertically shifted. **e,f,** Current-dependent SOT effective field ΔH_{SOT} of (**e**) Sn-BSTS/Fe_3GeTe_2 and (**f**) Sn-BSTS/ Fe_3GaTe_2 devices for M_z >0 and M_z <0. The solid lines in **c-f** represent the linear fits to the data.

11. Second harmonic measurements data for additional Sn-BSTS/Fe_3GeTe_2 and Sn-BSTS/Fe_3GaTe_2 devices

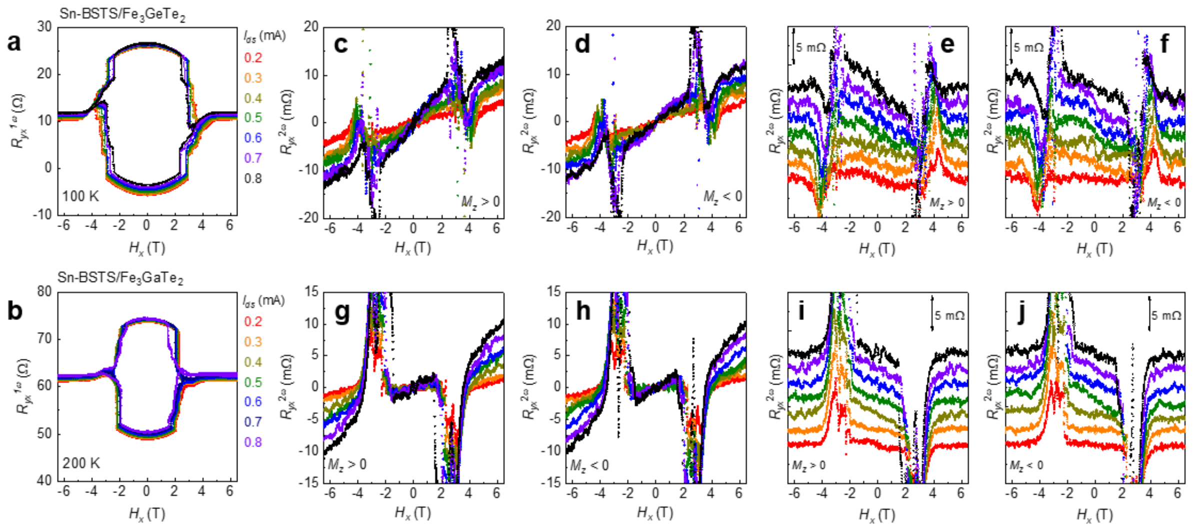

Figure S11. a,b The first harmonic Hall resistance ($R_{yx}^{1\omega}$) as a function of the in-plane magnetic field (H_x) parallel to the current direction at various current levels for Sn-BSTS/Fe_3GeTe_2 **(a)** and Sn-BSTS/Fe_3GaTe_2 **(b)** devices. **c,d,g,h,** The corresponding second harmonic resistances $R_{yx}^{2\omega}(H_x)$ for $M_z > 0$ and $M_z < 0$ in Sn-BSTS/Fe_3GeTe_2 **(c,d)** and Sn-BSTS/Fe_3GaTe_2 **(g,h)** devices. The same color codes are applied as in **a** and **b. e,f,i,j,** The second harmonic resistances $R_{yx}^{2\omega}(H_x)$ after subtracting the Nernst contribution at each current level for $M_z > 0$ and $M_z < 0$ in Sn-BSTS/Fe_3GeTe_2 **(e,f)** and Sn-BSTS/Fe_3GaTe_2 **(i,j)** devices. The data taken at different current levels are vertically shifted for clarity.

12. Current induced magnetization switching diagram for Sn-BSTS/Fe_3GeTe_2 devices

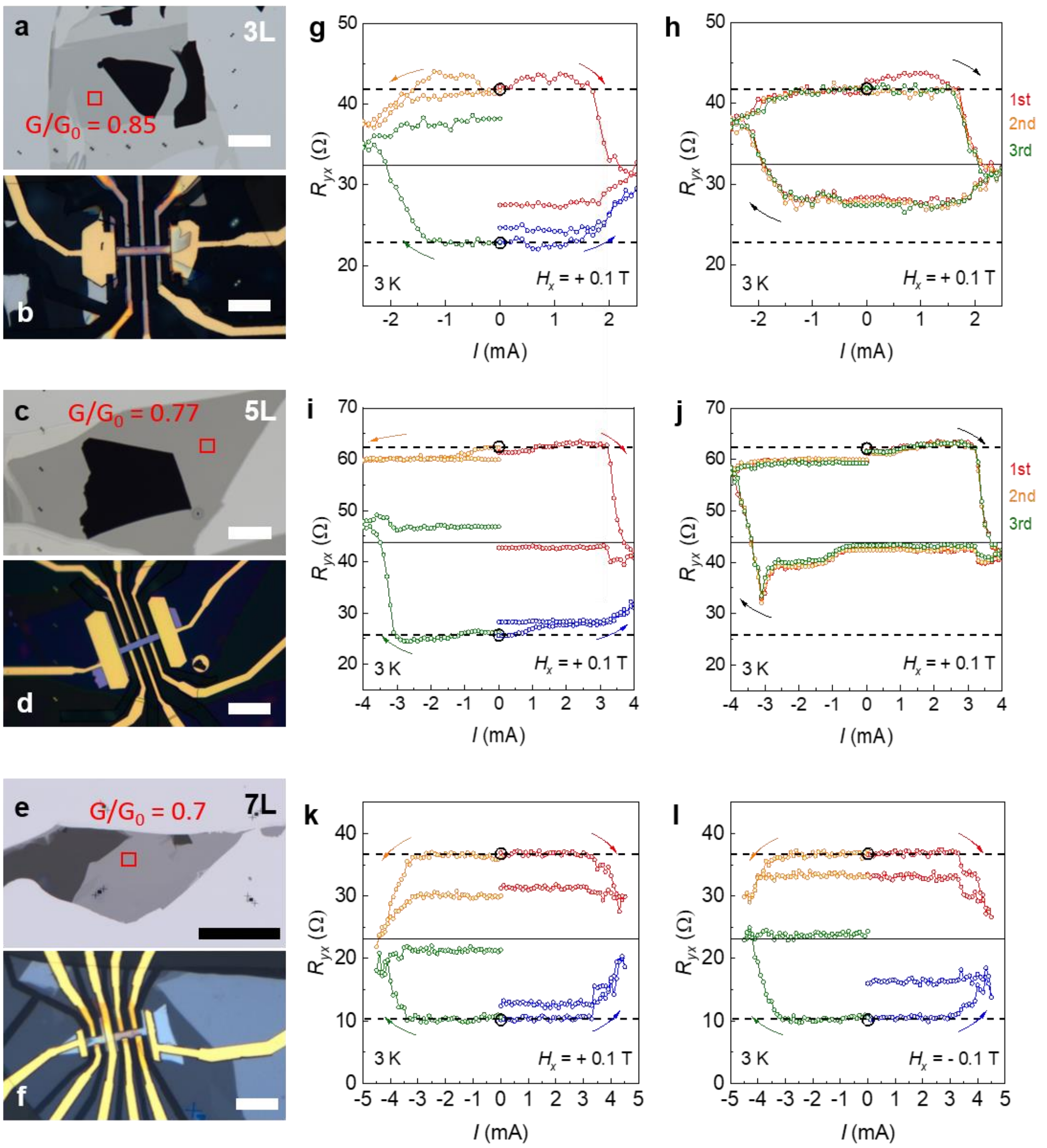

Figure S12. a-f, Transmitted optical images of Fe_3GeTe_2 nanoflakes **(a,c,e)** with thicknesses 3 layers (**a**), 5 layers (**c**) and 7 layers (**e**) determined by the normalized optical transmission G/G_0. The optical images of the corresponding devices after Hall bar patterning are shown in **b,d,** and **f**. Scale bars are 10 μm. **g-l,** Current-induced magnetization switching plots for SOT devices with different thicknesses of Fe_3GeTe_2 layers, 3L (**g,h**), 5L (**i,j**) and 7L (**k, l**). The switching Hall resistance (R_{yx}) curves with a single sweep of the applied current (I), starting from the initial states with fully saturated magnetization, were taken at 3 K under the in-plane magnetic field of H_x = 0.1 T for 3L and 5L devices (**g, i**) or at 100 K under H_x = +0.1 T (**k**) and -0.1 T (**l**) for the 7L device. The data with multiple current pulse sweeps are also obtained for 3L and 5L devices **(h,j)**. The Hall resistance for the fully saturated magnetization is indicated by the horizontal dashed lines. The current sweep direction is indicated by the arrows.
